# Millimeter wave direct-current transmission and reflection spectral data of some organic photo-responsive materials

**DOI:** 10.1016/j.dib.2019.104996

**Published:** 2019-12-17

**Authors:** Biswadev Roy, Taylor Knapp, Corrine Miller, Abay Gadisa, Harald W. Ade, Marvin H. Wu

**Affiliations:** a North Carolina Central University, Department of Mathematics & Physics, 1900 Concord St., Durham, NC, 27707, USA; b North Carolina School of Science & Mathematics, 1219 Broad St, Durham, NC, 27705, USA; c North Carolina State University, Department of Physics, Raleigh, NC, 27695, USA

**Keywords:** Photovoltaic, Organic, Millimeter, Transmission, Reflection, Voltage, Anomalous

## Abstract

Voltage data acquired after probe signal transmitted through the organic film and reflected off the film surface as a function of 0.36 mW millimeter wave signal frequency in the range 110–160 GHz. Five different organic photovoltaic (OPV) materials and one 95:5 blend produced at 2 spin rates are used. These materials are a) fluorinated 2-alkyl-benzol[d] [1–3]triazole (FTAZ), a high hole-mobility polymer used for transistors and photovoltaics, b) diketopyrrolopyrrole (DPP3T), an acceptor polymer used in field-effect transistors (FET), c) Y5(PffBT4T-2OD) film that possesses remarkable temperature controllable morphology, d) a neat conjugated polymer P3HT, Poly(3-(hexylthiophene-2,5diyl) film that is used in optoelectronic devices and as a conductive binder for Li-ion batteries, e) phenyl-C61-butyric acid methyl ester (PCBM) films and its soluble derivatives used as n-type organic semiconductors, and f) excitonic photovoltaic material 95%:5% donor-acceptor blend P3HT:PCBM produced by 2 different spin rates. Measurement of direct-current (dc) transmitted and reflected power (RF voltage signal) are measured using a newly developed continuous wave (CW) D-waveguide band probe (110–160 GHz) apparatus named time-resolved millimeter wave conductivity (TR-mmWC) [1]. Transmission and first surface reflection voltages are captured by a zero-bias Schottky barrier diode (ZBD) and converted to relevant dc voltages. Original voltage signal datasets attached with this can be utilized for photovoltaic, dielectric property estimation, and other semiconductor physics applications. A manually collected dataset of transmission and reflection coefficient at incident probe power level ∼0.9 mW for 95:5 P3HT:PCBM films produced at 2 different spin rates, and one separately only for the neat P3HT film are also presented here in tabular form.

**Table:** Specifications Table

Subject area	*physics*
More specific subject area	*Microwave/Millimeter wave dielectric properties of organic photo-responsive materials*
Type of data	*Floating point (E-notation); Raw data pertaining to transmission and reflection of millimeter waves at frequencies between* 110 GHz *and* 160 GHz *(resolution 0.*01 GHz*) are added as supplemental files. Some tabular datasets giving transmission coefficients and reflection coefficients at selected frequencies (based on manual observations) appear in*Table 1, Table 2*respectively*
How data was acquired	*Direct current output of transmitted and reflected RF power (voltage signal acquired through negatively polarized zero-biased Schottky detector) using antenna fed detector system*
Data format	*ASCII delimited; comma separated variables (CSV)*
Experimental factors	*Spin coated FTAZ and P3HT:PCBM (ratio- 95:5, produced by 2 different spin rates), Y5 created using optimum polymer solar cell morphology maintaining high crystallinity (excellent hole transport ability) with sufficient purity and reasonably small polymer domains; DPP3T n-type diketopyrrolopyrrole (DPP) – copolymer of DPP and terthiophene; extreme care is taken to keep probe beam free of interference in a free space setup using appropriate shielding*
Experimental features	*A recently developed time-resolved millimeter wave conductivity (TR-mmWC) setup that uses a highly coherent electrovacuum probe source (backward wave oscillator) operating in the D waveguide band (tunable between 107.35-*170 GHz*) with highly sensitive antenna fed Schottky barrier diode detector (negative polarity sensing) was used for transmission and reflection measurements. A gold mirror was used as a reference for reflection measurements. Direct current voltages in both transmission and reflection modes were read across an inductor of a bias-tee attached to the Schottky detector diode. The voltage datasets were collected using a Keithley 2782 digital multimeter. Each CSV output file structured as sweeper probe frequency, reference voltage (mean of 30 samples), standard deviation of the reference voltages, transmitted through sample/reflected off sample surface voltage (mean of 30 samples), and standard deviation of transmitted/reflected voltages of the sample between 110 and* 160 GHz *at a frequency resolution of 0.*01 GHz*. Anomalous data clusters/frequency points were flagged in the plots shown below and pointed out in respective figure captions for users.*
Data source location	*All data were collected at the TR-mmWC facility, microwave laboratory, located in Mary M. Townes Science Building of the School of Arts & Sciences, North Carolina Central University (NCCU) located at Durham, North Carolina. Lat./Lon.* 35.97° N/78.89° W
Data accessibility	https://data.mendeley.com/datasets/fv632kj9r5/5
Related research article	Biswadev Roy, Charles R. Jones, B. Vlahovic, Harald W. Ade and Marvin H. Wu, “A time-resolved millimeter wave conductivity (TR-mmWC) apparatus for charge dynamical properties of semiconductors”, Rev. Sci. Instrum. published Vol. 89, 104704 https://doi.org/10.1063/1.5026848*(2018)* [1].

**Table:** 

**Value of the Data** • Reflection and transmission voltage data as a function of millimeter wave frequency can be used to evaluate the basic dielectric property of the materials and could be used for developing its relationship with respective morphologies. Probe frequencies in the datasets pertain to the higher end of millimeter wave domain and close to the 5th generation radio communication spectrum, 5G. • The data could be used for calculation of local energy dissipated in the organic cell. • Data on these low surface-energy semiconducting thin films can be used for calculation of radio exposure parameters when such organic electronic materials are used for biomedical/bioelectronic devices, biochemical sensors, drug delivery, and neural interfacing. • For the future, acquisition of signal phase measurements with same resolution/frequency step size is planned to estimate complex dielectric properties [2].

## 1 Data

**Fig. 1:**
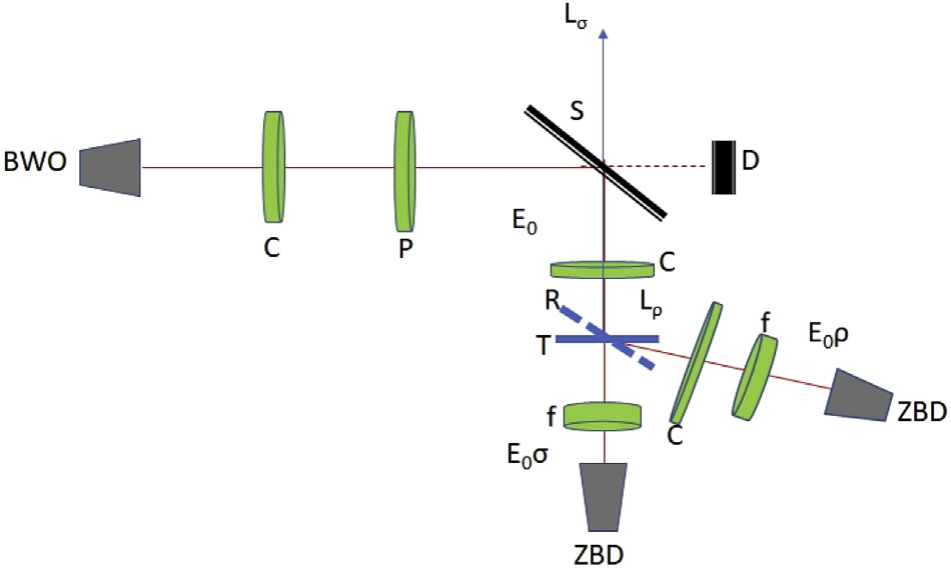
Shows the schematic for the experimental data acquisition for dc transmission (E_0_σ) and reflection (E_0_ρ) using the quasi-optical measuring channel that include TPX collimator lenses (C), wire grid polarizer (P), 2.5mm focusing lens (f), Mylar beam-splitter (S), beam dump(D), and zero-bias Schottky barrier diodes (ZBD). Backward wave oscillator (BWO) is swept using Labview 2017 and ZBD d.c. voltage data are processed and acquired using Keithley digital multimeter at sweep delay of 500 ms.

Photosensitive organic sample films of size 1″ x 1” (a few μm thick) spun on glass slides with various thicknesses are used for collecting transmitted and reflected voltages. The BWO probe source for transmission and reflection spectrum acquisition operated at 30% power level (10 mW) and a suitable beam splitter helps illuminate sample with a 2.8 mm diameter spot size and a power level ∼0.3 mW. Incident light on sample is part reflected and part transmitted. For the case of transmission, radiation of the probe signal is normally incident on the sample and for reflection, the probe beam is incident at 65.4° from the normal of the sample surface. If we consider E_0_ as the incident amplitude on approximately few μm thick organic sample mounted on glass substrate approximately 990 μm thick, we measure the reflected power off the sample (as ZBD voltage response) and also the transmitted power through the sample that includes the effect of multiple reflections through the glass substrate. A schematic of the experimental arrangement is shown below in Fig. 1.

For the data given here we have collected voltages only signifying real part of reflection and transmission coefficients and the spectra are shown at a frequency resolution 100 MHz. Following 4 steps are used for acquisition of transmission/reflection voltage response data:

1. Collect free-space voltage (E_0_) spectral response of ZBD by sweeping BWO between 110 and 160 GHz with resolution of 100 MHz and store data along with 10-sample standard deviation profile obtained from digital multimeter.
2. Mount sample in transmission mode making the BWO beam perpendicular to sample surface (T) shown in Fig. 1 and repeat the same sweeping procedure as in step 1 and store E_0_σ data obtained from multimeter.
3. Rotate sample holder by 24.6° and insert high quality gold mirror as shown in position R (dotted line) and run step 1 to collect the voltage spectrum of mirror reflection after sweeping the oscillator between 110 and 160 GHz, also record sample standard deviation.
4. Replace gold mirror with sample as shown in position R in Fig. 1 and repeat step 2 for collection of E_0_ρ (reflected voltage) data as function of frequency and corresponding standard deviation data from digital multimeter.

### 1.1 Through sample data

**Fig. 2:**
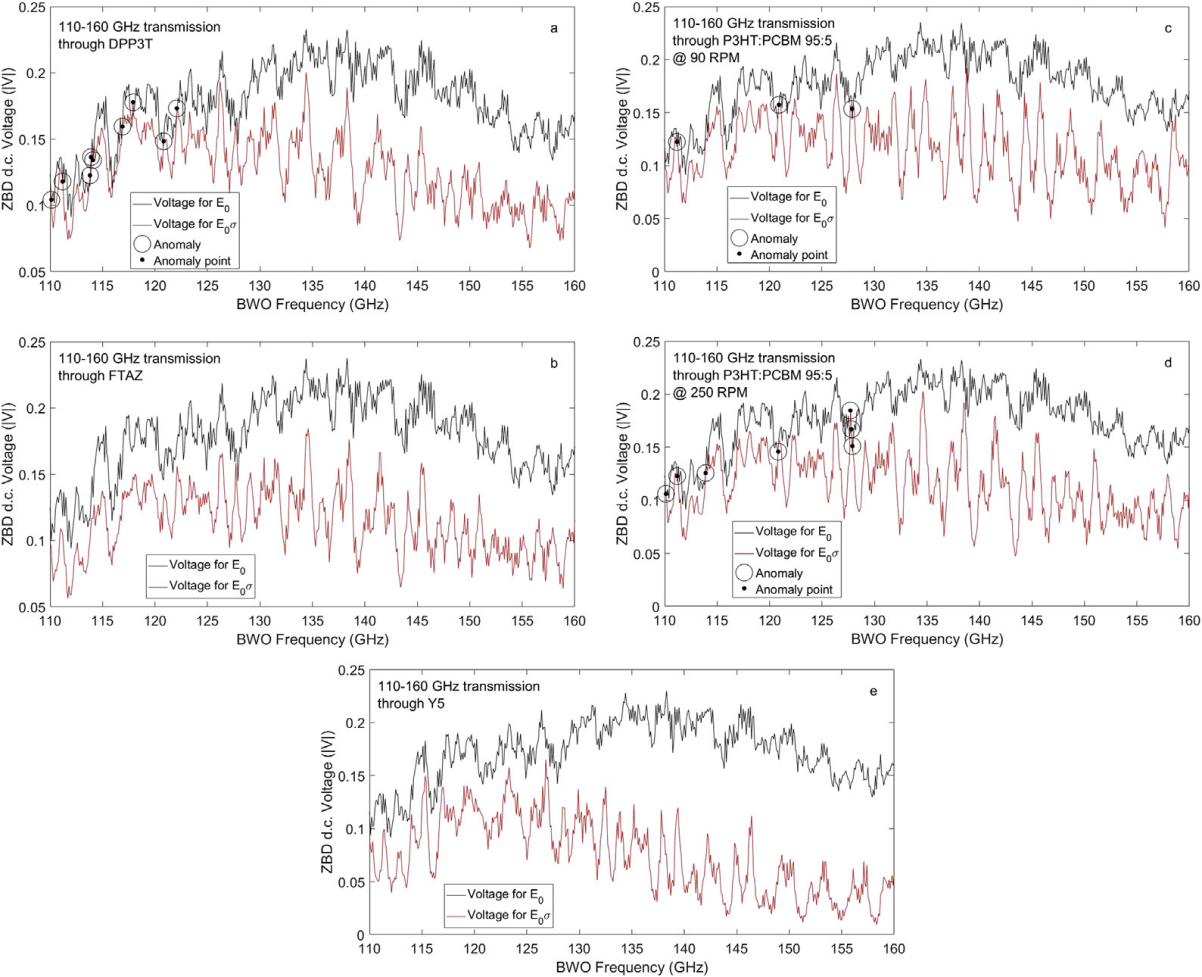
(a) Shows the transmitted voltage magnitude |V| spectra of the free space (in black) and assigned sample DPP3T (in red) for 0.32 mW millimeter wave power incident normally. Circle over data indicate anomalous data in through-sample transmission signal possibly due to standing wave/multiple reflections from 990 μm glass substrate. The DPP3T transmission data flagged pertain to 110.1, 111.2, 113.8, 113.9, 114.1, 116.9, 117.9, and 120.8 GHz. These data should be used with caution (b) Same as in (a) but for polymer FTAZ film [6,7], (c) Same as in (a) but for P3HT:PCBM (95%:5%) spun at 90 RPM with anomalous data flagged at frequencies: 111.2, 120.9 and 127.9 GHz, (d) Same as in (c) but P3HT:PCBM (95%:5%) spun at 250 RPM with anomalous transmission voltage data occurring at 110.1, 111.2, 113.9, 120.8, 127.7, 127.8 and 127.9 GHz respectively, these data should be used with caution, (e) Same as in (a) but for high crystalline sample Y5 from cold solution [8].

The transmission voltages shown here as obtained from the ZBD. An averaging of 10–30 samples are performed, and the data are stored in columnar fashion. The following Fig. 2(a–e) show the free-space and through-sample transmission data. Some of the sample transmission data show anomaly, where, through-sample voltages found to exceed free-space voltages for the same sample at same frequency and power level, especially in samples DPP3T [3,4] and P3HT:PCBM [5]. These anomalous voltages have been identified precisely and marked with a circle around the data points. Exact frequencies of these questionable transmission data were collected and are also given in Fig. 2 caption below.

### 1.2 Manually collected P3HT:PCBM and P3HT (neat) transmission coefficient

At 70% power level of the backward wave oscillator the probe beam is split and resulting ∼0.9 mW beam is transmitted through P3HT:PCBM and P3HT (neat) [9] samples spun at 250 rpm and at 90 rpm respectively. Detector voltages were collected for each measurement cycle in frequency range 110–165 GHz. Table 1 below has the E_0_σ/E_0_ data that were finalized after multimeter registered voltages stabilized in the first 15 seconds.

**Table 1:** Millimeter wave transmission coefficient computed using manually collected d.c. transmission voltages using higher oscillator power level (70%) and using reference E_0_ for each frequency.

P3HT:PCBM Sample	110 GHz	120 GHz	130 GHz	140 GHz	150 GHz	160 GHz	165 GHz
99:1 spun at 250 rpm	0.858561	0.884393	0.833656	0.66899	0.783113	0.817276	0.868376
95:5 spun at 250 rpm	0.828784	0.88632	0.83559	0.674216	0.781457	0.817276	0.870085
80:20 spun at 250 rpm	0.813896	0.8921	0.839458	0.75784	0.624172	0.855482	0.873504
50:50 spun at 250 rpm	0.82134	0.895954	0.829787	0.681185	0.778146	0.850498	0.859829
P3HT only Neat spun at 250 rpm	0.846154	0.888247	0.827853	0.655052	0.788079	0.883721	0.859829
99:1 spun at 90 rpm	0.277916	0.545279	0.638298	0.620209	0.703642	0.777409	0.82735
95:5 spun at 90 rpm	0.868486	0.888247	0.823985	0.656794	0.796358	0.880399	0.870085
80:20 spun at 90 rpm	0.873449	0.897881	0.839458	0.679443	0.783113	0.80897	0.863248
50:50 spun at 90 rpm	0.848635	0.915222	0.864603	0.703833	0.786424	0.805648	0.864957
P3HT only Neat spun at 90 rpm	0.880893	0.890173	0.82205	0.648084	0.798013	0.887043	0.854701

**Table 2:** Millimeter wave reflection coefficient computed using manually collected d.c. reflected voltages at fixed (150 GHz) frequency and at 30% power level (0.32 mW) using reference E_0g_ (mirror reflection) for each sample.

P3HT:PCBM Sample	99:1 spun at 250 rpm	95:5 spun at 250 rpm	80:20 spun at 250 rpm	50:50 spun at 250 rpm	P3HT Neat spun at 250 rpm	99:1 spun at 90 rpm	95:5 spun at 90 rpm	80:20 spun at 90 rpm	50:50 spun at 90 rpm	P3HT Neat at 90 rpm
Reflection Coefficient at 150 GHz (0.32 mW)	0.504	0.413	0.428	0.406	0.496	0.428	0.428	0.353	0.436	0.157

### 1.3 Sample reflection data

**Figure 3:**
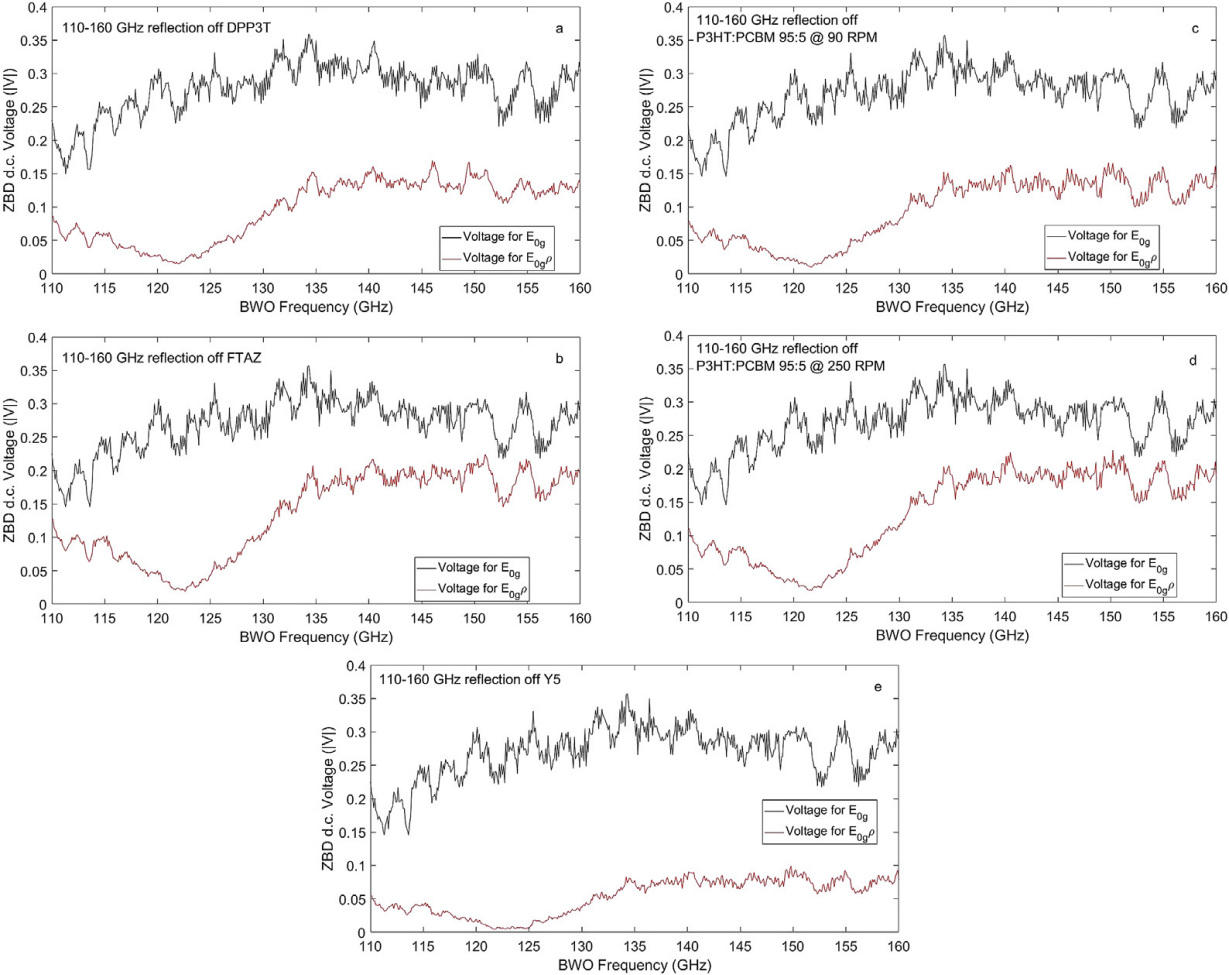
(a) Shows the reflected voltage magnitude |V| spectra of the highly polished mirror (black) and assigned sample DPP3T (in red) for 0.32 mW millimeter wave power incident at 65.4° on mirror/sample surface. (b) Same as in (a) but for polymer FTAZ film, (c) Same as in (a) but for P3HT:PCBM 95:5 spun at 90 RPM, (d) Same as in (c) but P3HT:PCBM spun at 250 RPM, (e) Same as in (a) but for high crystalline sample Y5 from cold solution.

The sample reflection data are shown here. For the purpose of reference, we use a highly polished mirror and label the field as E_0g_. An averaging of 10–30 samples are performed and the data are stored in columnar fashion. The following Fig. 3(a–e) show the ZBD acquired and multimeter averaged mirror reflection and sample reflection d. c. voltage datasets.

### 1.4 Manually collected P3HT:PCBM and P3HT (neat) reflection coefficient

Each of the P3HT:PCBM and P3HT neat samples that were produced at different spin rates were manually placed on sample holder one at a time, probe beam maximized for power and the voltages were recorded once with sample, and another time without the sample. Absolute care was taken so as not to alter the quasi-optical settings of the apparatus while these observations were noted.

## 2 Experimental design, materials, and methods

**Fig. 4:**
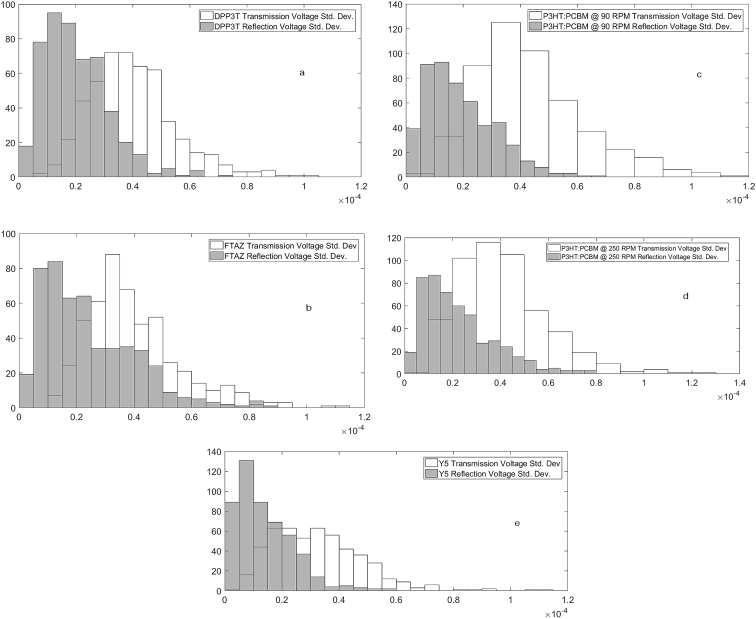
(a)–(e) shows the histogram of voltage standard deviations obtained while running the experiment in transmission (white face) and reflection (gray face) modes.

All the polymers and polymer:fullerene (1:1) solutions were prepared by dissolving in a chlorobenzene solvent and stirred in a nitrogen-filled glove box. The films were spin cast or drop cast and dried in the glove box before they were taken out for characterizations. For spin-cast films, thicknesses were varied by changing spin speeds. Voltage standard deviations maxima were found to be in range 0.1–0.12 mV for transmission cases and around 0.06–0.12 mV for the reflection datasets as recorded. Fig. 4 provide the standard deviation for the transmission and reflection cases for each sample.
